# Author Correction: Bacterial detoxification of plant defence secondary metabolites mediates the interaction between a shrub and frugivorous birds

**DOI:** 10.1038/s41467-026-73042-y

**Published:** 2026-07-28

**Authors:** Beny Trabelcy, Nimrod Shteindel, Maya Lalzar, Ido Izhaki, Yoram Gerchman

**Affiliations:** 1 Department of Evolutionary and Environmental Biology, Faculty of Natural Sciences, University of Haifa, Haifa, 3498838 Israel; 2 Bioinformatic Unit, University of Haifa, Haifa, Israel; 3 Oranim College, Kiryat Tivon, 3600600 Israel

**Keywords:** Ecological networks, Chemical ecology, Plant evolution, Microbial ecology

Correction to: *Nature Communications* 10.1038/s41467-023-37525-6, published online 31 March 2023

In the version of this article initially published, there were errors in Fig. 1c where colors in each column were interchanged, now corrected to present blue on the left, yellow on the right. In the second-to-last paragraph of the Discussion, in the text now reading “Furthermore, *P. agglomerans* can remediate the physiological consequences of BITC exposure,” the species “*P*. *xanthopygos*” was originally listed. The figure and text are now amended in the HTML and PDF versions of the article.

